# Lymphovascular Invasion (LVI) Correlates with Systemic Immune-Inflammation Index (SII) in Adenocarcinoma of the Gastroesophageal Junction (AEG): Implications for Prognostic Stratification

**DOI:** 10.3390/cancers17162604

**Published:** 2025-08-08

**Authors:** Gerd Jomrich, Winny Yan, Dagmar Kollmann, Ivan Kristo, Benjamin Fallmann, Hannah Puhr, Aysegül Ilhan-Mutlu, Marlene Hollenstein, Reza Asari, Christian Sebesta, Sebastian F. Schoppmann

**Affiliations:** 1 Department of General Surgery, Gastroesophageal Tumor Unit-Comprehensive Cancer Center (CCC), Medical University of Vienna, 1090 Vienna, Austria; gerd.jomrich@meduniwien.ac.at (G.J.); winny.yan@meduniwien.ac.at (W.Y.); dagmar.kollmann@meduniwien.ac.at (D.K.); ivan.kristo@meduniwien.ac.at (I.K.); reza.asari@meduniwien.ac.at (R.A.); 2 Center for Medical Data Science, Institute of Medical Statistics, Medical University of Vienna, 1090 Vienna, Austria; benjamin.fallmann@meduniwien.ac.at; 3 Department of Medicine 1, Gastroesophageal Tumor Unit-Comprehensive Cancer Center (CCC), Medical University of Vienna, 1090 Vienna, Austria; hannah.puhr@meduniwien.ac.at (H.P.); aysegul.ilhan@meduniwien.ac.at (A.I.-M.); 4 Department of Laboratory Medicine, Medical University of Vienna, 1090 Vienna, Austria; marlene.hollenstein@meduniwien.ac.at; 5 2nd Medical Department, Klinik Donaustadt, Science Center Donaustadt, Vienna Cancer Center (VCC), 1220 Vienna, Austria; christian.sebesta@gesundheitsverbund.at

**Keywords:** adenocarcinoma of the gastroesophageal junction, prognostic parameter, inflammation, lvi, neoadjuvant therapy

## Abstract

Adenocarcinoma of the gastroesophageal junction (AEG) is an aggressive cancer with rising incidence and poor long-term survival despite multimodal treatment. Reliable preoperative prognostic markers are lacking. Methods: The study is based on data from a prospectively maintained institutional database, which was retrospectively analyzed. In total, 211 patients were analyzed who underwent curative resection for AEG to evaluate the association of SII and LVI and their combined prognostic value. Results: LVI was present in 45% of patients and was significantly associated with higher median SII values compared to patients without LVI (943 vs. 652; *p* < 0.001). Both high SII and the presence of LVI were independently associated with worse overall survival (*p* < 0.001 for both). In patients treated with primary surgery (65%), the combined presence of high SII and LVI identified a subgroup with particularly poor prognosis (pseudo-R^2^ increased from 0.451 to 0.524; likelihood-ratio test *p* < 0.001). Among patients who received neoadjuvant therapy (NT) (35%), SII remained a strong prognostic factor (pseudo-R^2^ = 0.432), while LVI alone was not statistically significant (*p* = 0.135), and its addition to the SII model did not improve prognostic performance (*p* = 0.377). Conclusions: The combined assessment of SII and LVI may improve prognostic stratification in AEG, especially in patients undergoing upfront surgery. These findings suggest that combined assessment of SII and LVI enhances prognostic stratification, particularly in patients treated with upfront surgery, and may aid personalized treatment and follow-up planning in AEG. To the best of our knowledge, this is the first study investigating the association of SII and LVI in AEG.

## Simple Summary

Adenocarcinoma of the gastroesophageal junction (AEG) is an aggressive cancer with poor survival despite curative surgery. Reliable and simple prognostic markers are urgently needed to improve patient management. This study examines two key factors: systemic immune-inflammation Index measured in blood, tumor invasion into small blood and lymphatic vessels (lymphovascular invasion, LVI). Although both have individually been associated with worse outcomes, their combined relationship in AEG patients has not been explored. Understanding this link may improve personalized treatment strategies, help identify patients needing closer follow-up or intensified therapy, and guide future research on AEG therapies.

## 1. Introduction

Esophageal cancer ranks as the sixth most common cause of cancer-related mortality worldwide. In recent decades, the incidence of adenocarcinoma of the gastroesophageal junction (AEG) has risen significantly [1,2,3]. Patients are often diagnosed at advanced or metastatic stages, due to its asymptomatic nature at early stages [1]. Despite progress in multimodal treatment strategies, the prognosis for AEG remains poor with a 5-year survival rate of 20% in the Western world [4]. Even with the combination of novel systemic therapies and surgery, around 50% of patients experience disease recurrence. Currently, prognostic assessments rely on postoperative histopathological factors such as tumor and lymph node staging, degree of differentiation, and resection margin status [5].

Inflammation has been recognized as an important role in the development and progression of cancer. The systemic immune-inflammatory response of the host has appeared to be a key determinant of tumor behavior und prognosis. Several inflammatory markers – such as the neutrophil–lymphocyte ratio (NLR) [6,7], platelet–lymphocyte ratio (PLR) [8], the Glasgow prognostic score (GPS) [9], the fibrinogen and albumin ratio (FAR) [10] and the systemic immune-inflammation index (SII) [11] - demonstrated prognostic relevance in various solid tumors, including AEG. Among these, the SII based on neutrophil, lymphocyte, and platelet counts have emerged as a robust integrative marker of tumor-promoting inflammation and host immune competence [12,13]. Its prognostic relevance has been demonstrated in various solid tumors, including hepatocellular [14], pancreatic [15], breast [16] and AEG [10], reflecting the balance between host inflammation and immune status.

Moreover, lymphovascular invasion (LVI) refers to the presence of tumor cells within lymphatic vessels, representing crucial early steps in the metastatic cascade. Tumor-associated lymphatic vessels are the main path for cancer cells to disseminate to regional lymph nodes and distant organs, thereby contributing to systemic disease and poor prognosis among various malignancies [17,18,19]. The process of tumor-induced lymphangiogenesis has been linked to an increased risk of metastasis and reduced survival [20]. With the advent of specific lymphatic endothelial markers, it is now possible to distinguish lymphatic from blood microvessels, enabling more precise analysis of lymphatic spread mechanisms. LVI has consistently emerged as a marker of aggressive disease biology. Its presence is associated with poor survival, increased recurrence risk, and a higher likelihood of nodal metastasis in esophageal adenocarcinoma [21]. While the prognostic significance of both SII [15] and LVI [22] in patients with AEG has been well established, their potential association has not yet been systematically explored.

## 2. Materials and Methods

This study included patients who underwent curative resection for locally advanced AEG between January 1992 and April 2016. Data were obtained from a prospectively maintained database at the Department of Surgery at the Medical University of Vienna. Clinical, pathological, and follow-up data were retrieved from an institutional surgical oncology registry that has been prospectively maintained. This database includes patients who underwent curative resection for AEG between January 1992 and April 2016. All data were recorded at the time of treatment and during routine follow-up, ensuring systematic and standardized documentation. For the present study, these prospectively collected data were analyzed retrospectively. Patients were excluded if they were presented with distant metastases at the time of surgery, positive resection margins, missing preoperative platelet, neutrophil, and lymphocyte counts, or malignancies other than AEG. At the time of blood sample collection, which was performed either before the start of NT or prior to surgery in patients undergoing primary surgical treatment, none of the patients showed signs of fever or any type of active infection or chronic inflammatory disease. The study was approved by the Ethics Committee of the Medical University of Vienna, in accordance with the Declaration of Helsinki, histopathological, and laboratory data of the patients were retrospectively reviewed and collected from the local database and patient records.

The SII is calculated as platelet count × neutrophil count divided by lymphocyte count, integrates three peripheral blood parameters and reflects the balance between host inflammation and immune response. Clinical tumor staging was performed using the TNM classification of the Union for International Cancer Control (UICC), 7th edition [23]. All patients were discussed on a multidisciplinary tumor board prior to treatment. Patients receiving NT were treated according to the standards of the Comprehensive Cancer Center of the Medical University of Vienna. Therapy models included either oxaliplatin/capecitabine-based or cisplatin/5-fluorouracil-based protocols, combined with radiation doses ranging from 42 to 46 Gray. Tumor regression grade (TRG) in response to NT was classified based on the system defined by Mandard et al. [24].

Tumor localization at the gastroesophageal junction was classified according to the system proposed by Siewert and Stein [25]. The choice of surgical approach, either abdominothoracic en-bloc esophagectomy or transhiatal extended gastrectomy, depended on the primary tumor location. For the survival analysis, the cutoff for the lymph node ratio was determined to be 0.03, based on established thresholds reported in previous literature [26]. However, the statistical analysis uses the ratio directly. All patients were followed up regularly through physical examinations, tumor marker assessments, and CT scans. Follow-ups were conducted every three months during the first two years and then every six months up to five years after surgery.

### 2.1. Immunohistochemistry

The immunohistochemistry was performed on 3 μm thick paraffin sections, applying the Benchmark Ultra immunostainer (Ventana) according to the manufacturer’s instructions. Podoplanin expression was detected with the mouse moncolonal anti-podoplanin antibody D2-40 (catalog number 760–4395, Venatana, Tucson, AZ, USA, ready to use). In three randomly chosen cases, additional staining was performed for vimentin using antibody clone SP20 (Thermo Fisher Scientific, Fremont, CA, USA; dilution 1:300) and for alpha-smooth muscle actin (α-SMA) with antibody clone 1A4 (DAKO, Glostrup, Denmark; dilution 1:200). Two independent observers assessed all slides. In cases of disagreement, the slides were re-evaluated using a multiheaded microscope.

A specimen was scored as positive for podoplanin expression in CAFs (CAF+), when ≥10% of the fibromatous tumor stroma showed a distinct staining reaction [21]. Lymphatic vessels served as internal positive control. Furthermore, LVI of tumor cells in primary tumors was scored as positive, if tumor cells were seen in at least one podoplanin decorated vascular space. A specimen was considered as showing podoplanin expression in tumors cells if positive cytoplasmic staining reaction was seen, regardless of percentage or intensity.

Samples were classified as positive for podoplanin expression in cancer-associated fibroblasts (CAF+) if ≥10% of the fibrmatous tumor stroma exhibited distinct staining [9]. Lymphatic vessels served as internal positive controls. LVI by tumor cells in primary tumors was considered present if tumor cells were observed within at least one podoplanin-positive vascular space. Podoplanin expression in tumor cells was considered positive if a cytoplasmic staining reaction was observed, regardless of staining intensity or the proportion of positive cells.

### 2.2. Statistical Analysis

Mean, standard deviation, median, and quartiles were used to describe continuous variables, whereas counts and percentages were used to summarize categorical variables. For dates, the earliest, median, and latest date were given. The survival function of the patients was estimated using the Kaplan–Meier estimator. The association between the LVI and SII was tested using Wilcoxon rank-sum test, and the probability of superiority for those values was calculated for all patients and those who did and did not receive NT, respectively. Cox regression models were fitted for the two primary variables and the additionally used explanatory variables. For each subgroup of patients with or without NT, using the primary variables as a base, the effect of inclusion of further covariates was evaluated based on the Akaike information criterion (AIC). The best models according to the AIC were then listed. For the univariate models of the primary variables and the multivariate models, the Brier score over time was plotted and the Integrated Brier Score (IBS) was calculated. For additional evaluation of their influence over time, the effects of the primary variables on survival were also plotted as Kaplan–Meier curves. The SII was dichotomized at a cutoff of 750, which corresponded approximately to the median value in the study cohort allowing a balanced group comparison. All calculations were performed using the statistical software R (version 4.4.2).

## 3. Results

**Figure A1:**
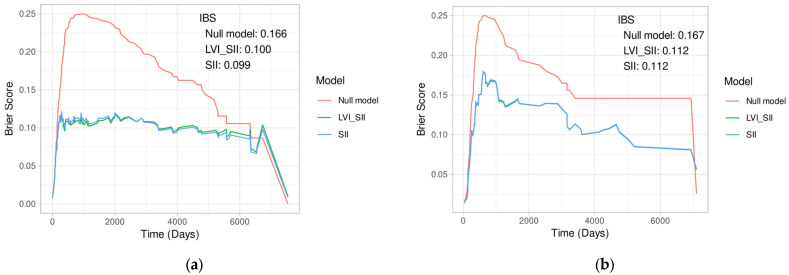
(**a**) Brier Score over time of the best multivariate models of primarily resected patients (**b**) Brier Score over time of the best multivariate models of patients with NT.

In total, 211 patients were analyzed who underwent surgery for resectable AEG. Of these, 74 patients (35%) received NT while 137 (65%) underwent primary surgical resection without prior treatment. The mean age at the time of surgery was 65 years (±11), and the majority of patients were male (*n* = 172, 82%), with 39 female patients (18%). Descriptive statistics of various variables of interest, overall and itemized by NT, can be found in Table 1 and Table A1. The mean SII for patient with positive LVI was 943, versus a score of 652 in patients without LVI. LVI and SII were found to be strongly linked, with a *p*-superiority of 0.27 (95% CI: (0.21, 0.34)), as shown in Table 2. For patients who had received NT, this value was 0.34 (0.24, 0.47), compared to 0.23 (0.17, 0.31) for primarily resected patients. Fitting Cox regression models for just the primary variables found them to have a strong effect on survival (*p* < 0.001), except for LVI on the subgroup of those who did receive NT (*p* = 0.135). The resulting model coefficients can be found in Table 3, Table 4 and Table 5. The model selection showed that, although very different models were selected as best for primarily resected patients and patients with NT, no additional benefit could be seen for including the LVI. This result is further supported by the Brier score over time of those models, as seen in Figure A1.

For the additional Kaplan–Meier plots, patients were binned into groups according to their SII in two groups of about similar size. The plots seem to be split into main section and appendix.

### 3.1. Descriptive Statistics

Table 1 shows the baseline characteristics of all patients. LVI was histologically confirmed in 35% of patients. Most patients (59%) had stage III disease preoperatively. Postoperatively, 42% remained in stage III, while 28% were in stage II. The majority of patients had good functional status (ASA I–II: 87%; ECOG 0–1: 88%). Histologically, 55% of tumors were poorly differentiated (G3). Tumor location was predominantly AEG I (55%). Pathological lymph node metastases were present in 61% of patients. Additionally, Table A1 shows a descriptive summary of patients based on their LVI status.

**Figure 1:**
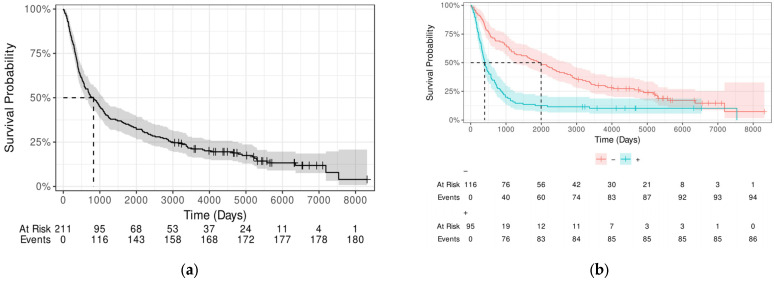
(**a**) Kaplan–Meier estimates survival rates for all patients (**b**) Kaplan–Meier estimates survival rates of patients by LVI (+ or −).

Using the Kaplan–Meier estimator plotted in Figure 1a, the results show that the median survival time for all patients was 827 days (95% CI: 585–1091). The 5-year survival rate was 34% (95% CI: 28–41%). A Kaplan–Meier plot differentiating by LVI status can be found in Figure 1b. For patients with negative LVI, the median survival rate was 1959.5 days (95% CI: 1236–2731), whereas the median survival rate for patients with LVI was 395 days (95% CI: 330–573). The 5-year survival was, for people without and with LVI respectively, 51.7% (95% CI: 43.4–61.7%) and 13.7% (95% CI: 8.2–22.7%).

### 3.2. Association Between SII and LVI

The statistical analysis using the Wilcoxon rank-sum test in Table 2 shows a significant association between SII and the presence of LVI (*p* < 0.001), with a *p*-superiority of 0.27 (95% CI: 0.21–0.34). Subgroup analyses revealed a slightly higher *p*-superiority among patients who received NT (0.34) than among primarily resected patients (0.23).

### 3.3. Cox Regression Models

In order to improve the legibility of the results, the SII was divided by 100 for all the models in this section.

#### 3.3.1. Models with SII and LVI

**Figure A2:**
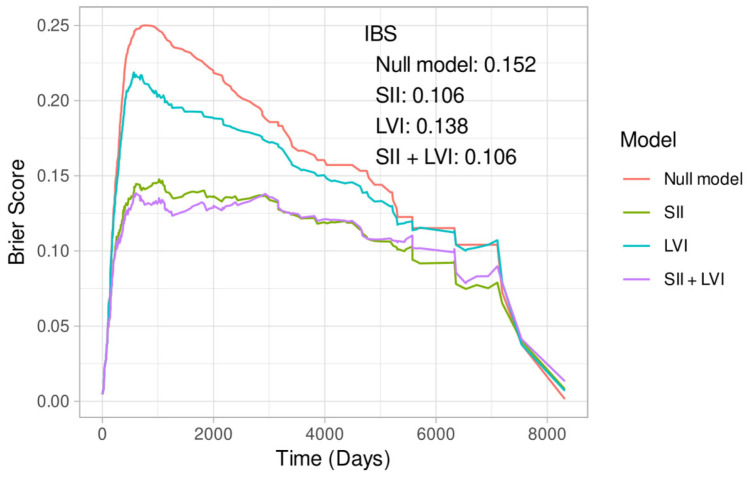
Brier Score over time of the different univariate models for all patients.

Initial models were fitted using only the primary variables LVI and SII. The results of these analyses are presented in Table 3. Both LVI and SII have a strong effect on the survival outcome, on their own as well as combined. Testing for proportional hazards using Schoenfeld residuals showed violations for both SII and LVI for the full population in the time period after 2000 days, when more than 75% of events already occurred. The hazard ratios should therefore be interpreted with caution. The *p*-values are also not adjusted for multiple testing. The given R^2^ Values are the pseudo-R^2^ by Cox and Snell. The addition of the Parameter LVI compared to using only SII was also found to be significant using a likelihood-ratio test (*p* < 0.001). Looking at the Brier score, and specifically the IBS, overall, the observed time range shows that this difference did not result in qualitative differences in the models prediction ability, as can be seen in Figure A2.

The Cox regression revealed that both the SII and LVI individually showed a strong association with overall survival in all patients. When both variables were combined in a model, their effect remained significant, indicating that each contributes independently to patient prognosis. Specifically, SII alone had a higher pseudo-R^2^ (0.441) compared to LVI alone (0.128), highlighting that SII captures substantial prognostic information. However, adding LVI to the model with SII further improved the model fit significantly (likelihood-ratio test *p* < 0.001), even if the predictive performance, measured by the Brier score and IBS, did not show major improvements (Figure A2). Regarding the subgroups, there can be seen differences between patients with and without NT. In the primarily resected patients (no NT), both SII and LVI remained strong and independent prognostic factors, and their combination significantly improved the model performance (likelihood-ratio test *p* < 0.001). Here, the pseudo-R^2^ increased noticeably when combining SII and LVI (from 0.451 to 0.524), indicating meaningful added prognostic value. In contrast, among patients who had received NT, SII remained a significant predictor of survival, while LVI alone did not reach statistical significance (*p* = 0.135). Furthermore, adding LVI to the SII model in the NT group did not significantly improve the model (likelihood-ratio test *p* = 0.377), and the increase in pseudo-R^2^ was minimal (from 0.432 to 0.438).

Overall, these findings suggest that SII and LVI combined captures substantial prognostic information and the additional prognostic value of LVI is particularly evident in patients who did not receive NT. In contrast, for patients treated with NT, SII alone appears to be sufficient for risk stratification.

**Figure 2:**
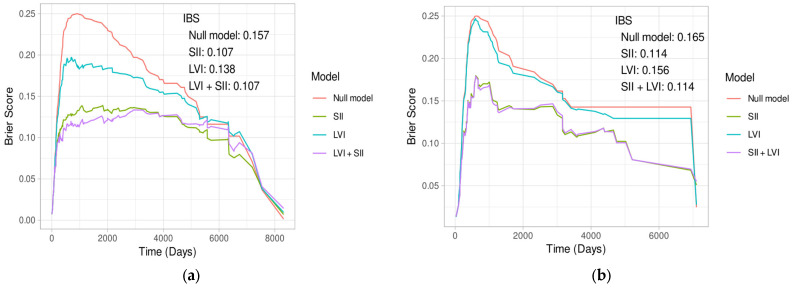
(**a**) Brier score over time of the different univariate models for primarily resected patients; (**b**) Brier score over time of the different univariate models for patients with NT.

The same models are also fitted for the subgroups of primarily resected patients and those that received NT. The results can be found in Table 4 and Table 5 respectively. As shown, comparing models that include LVI with those based solely on SII using a likelihood-ratio test showed a significant result (*p* < 0.001) in patients without NT, but not in those with NT (*p* = 0.377). Similarly, the Brier scores and IBS of the different models (Figure 2a,b) indicate no substantial difference in predictive performance.

#### 3.3.2. Other Univariate Models

In the univariate analysis, several factors showed significant associations with survival outcomes are shown in Table A2. UICC postoperative stage was a strong prognostic factor in the primarily resected group, with patients in stages II (HR = 3.10, *p* < 0.001), III (HR = 4.93, *p* < 0.001), and IV (HR = 3.27, *p* = 0.006) demonstrating worse survival compared to stage I. In contrast, postoperative stage did not significantly affect survival in the NT group. The lymph node ratio was also a significant factor in the primarily resected group (HR = 22.4, *p* < 0.001), indicating that a higher lymph node ratio was associated with poorer survival. It remained significant in the NT group, though with a smaller effect size (HR = 3.71, *p* = 0.004). Finally, the surgical technique (specifically, a two-chamber approach) was found to be a significant predictor in the primarily resected group (HR = 0.68, *p* = 0.036).

#### 3.3.3. Model Selection with Additional Variables

Model selection based on the Akaike information criterion (AIC) was used to identify the best multivariate survival models incorporating SII, with or without LVI, alongside additional clinicopathological variables. To decide on relevant covariates for the model, a model with SII or LVI + SII and any combination of the following variables for both the subgroups was fitted: sex, age at OP, pUICC, pT, pN, G, tumor type, surgical technique, ASA and ECOG.

For only the subgroup of patients with neoadjuvant therapy, additional variables were added consistently: Mandard score and NT. The AIC was then calculated for each of those models. To ensure comparability between the models, only patients where all those variables were present could be used. This resulted in a sample of 129 primarily resected patients and 72 patients who received NT. The three best-scoring models for each group are listed in Table 6 and Table 7.

For both groups, the best model according to the AIC uses the same additional variables regardless of the inclusion of LVI, although what variables are selected differs in the two patient subgroups. For both groups, however, the best model with LVI has a slightly worse AIC than the one without LVI. The values of the parameters for the best models according to this selection can be found in Table 8 and Table 9.

For both patient subgroups, patients without (Table 8) and with NT (Table 9), the best-fitting models consistently did not include LVI. In fact, models excluding LVI often had slightly better AIC scores. Predictive accuracy, as measured by Brier scores and IBS, also showed no significant improvement with LVI inclusion, suggesting limited incremental value for this variable when accounting for other covariates.

### 3.4. Survival Analysis

**Figure 3:**
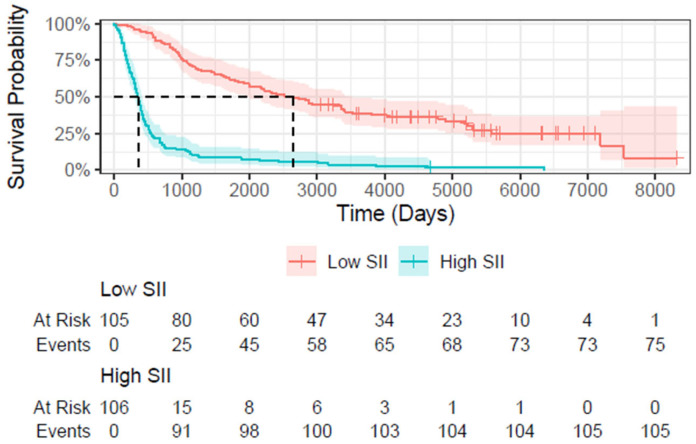
Kaplan–Meier estimates of survival for different SII values.

Since proportional hazards cannot be fully assumed for the models, an alternative way to assess the influence of the parameters on survival has been provided using Kaplan–Meier curves. The SII variable has been divided in two groups in those with a score lower/higher than 750. This cutoff was chosen to obtain groups with about equal number of patients. The resulting plot comparing only patients with different SII Scores is shown in Figure 3.

**Figure 4:**
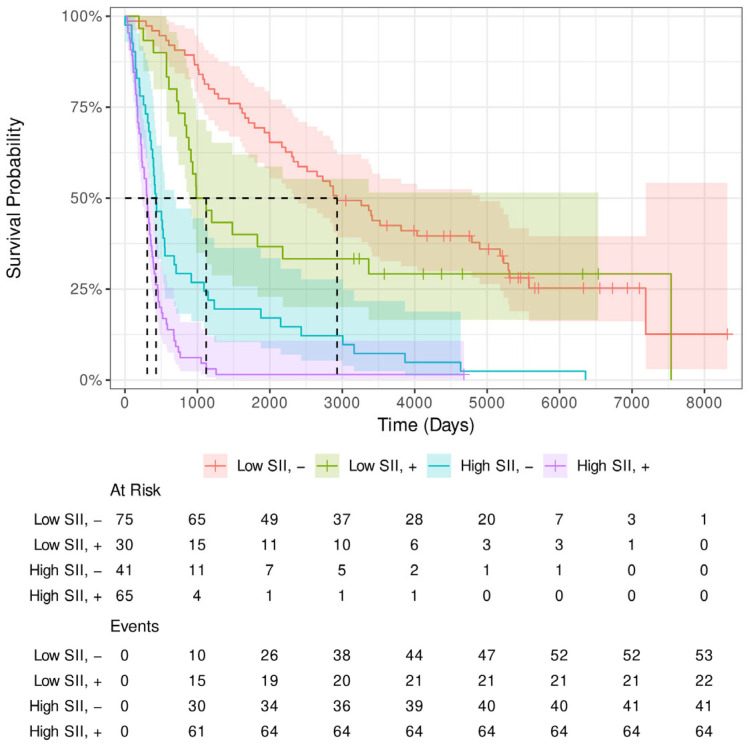
Kaplan–Meier estimates of survival for different combinations of LVI (+, −) and SII (low, high).

**Figure A3:**
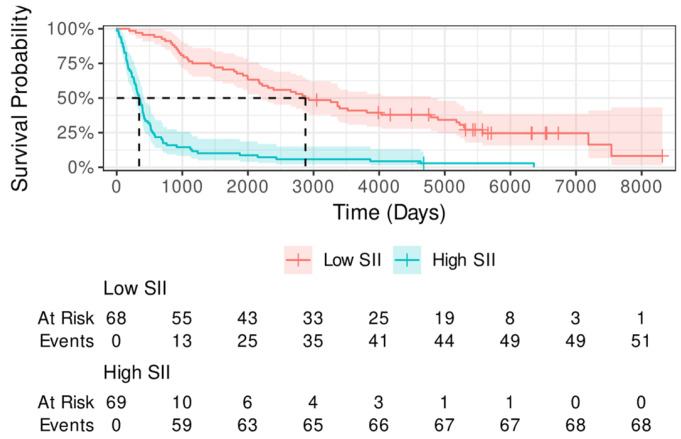
Kaplan–Meier estimates of survival for different SII values for patients who did not receive NT.

**Figure A4:**
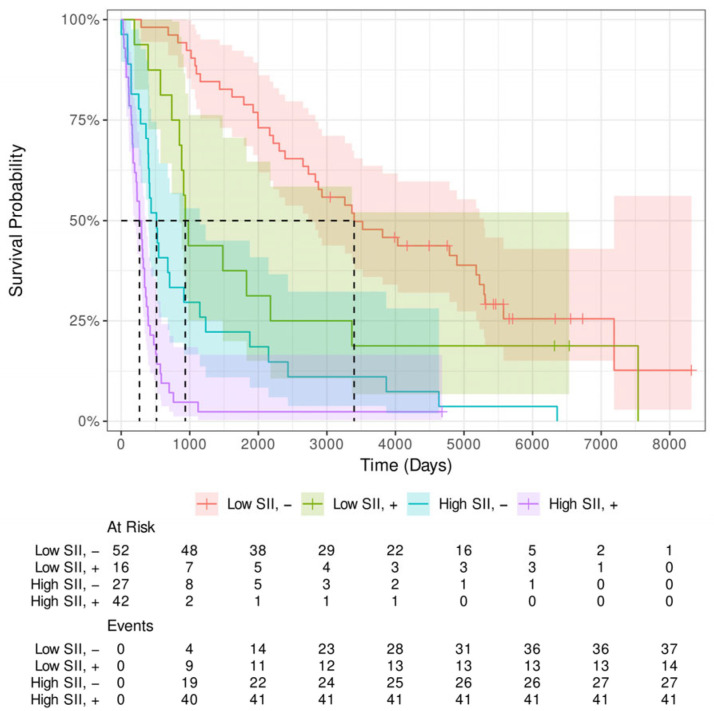
Kaplan–Meier estimates of survival for different combinations of LVI (+, −) and SII (low, high) for patients who did not receive NT.

**Figure A5:**
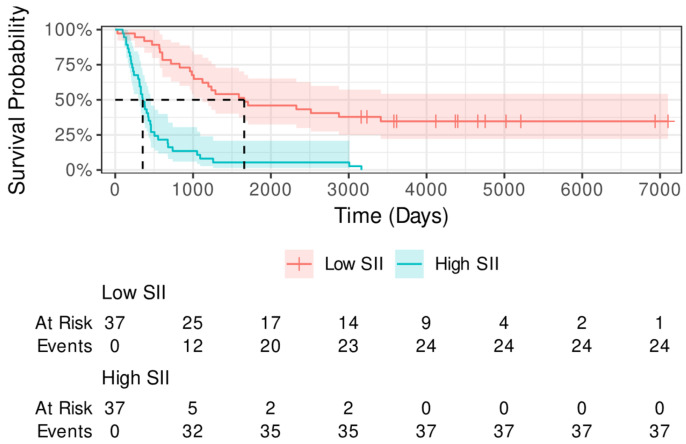
Kaplan–Meier estimates of survival for different SII values for patients who received NT.

**Figure A6:**
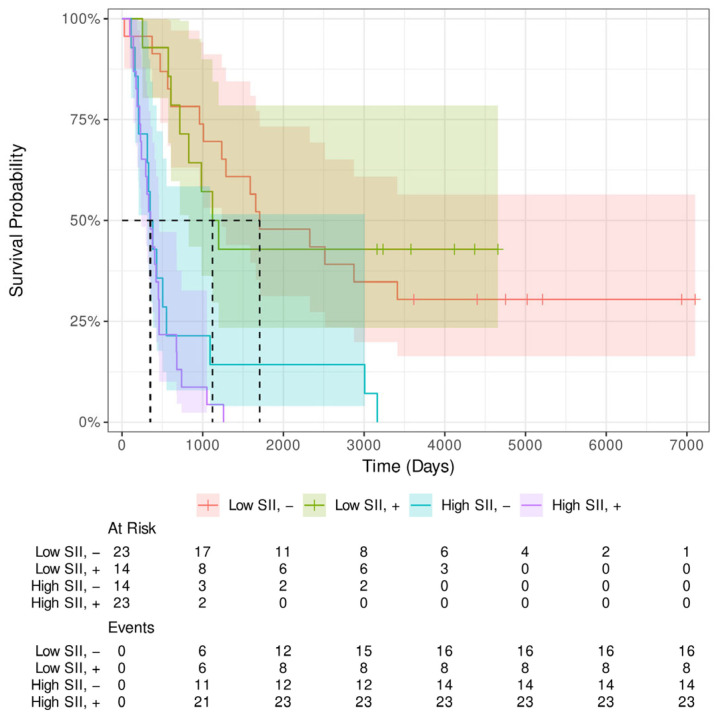
Kaplan-Meier estimates of survival for different combinations of LVI (+, −) and SII (low, high) in population that received NT for different combinations.

A similar plot considering additionally the LVI can be found in Figure 4. The same curves are also plotted using only patients who did not receive NT shown in Figure A3 and Figure A4 and those that did receive NT shown in Figure A5 and Figure A6.

The Kaplan–Meier survival analysis provides valuable insights into the prognostic significance of the SII and LVI in patients, both with and without NT. SII has shown to be a strong and independent predictor of survival in both patient subgroups. In the cohort of patients who did not receive NT, high SII was associated with poorer survival outcomes compared to low SII. This trend was observed across all time points, indicating that elevated SII correlates with worse prognosis. Similarly, in patients who underwent NT, high SII was also linked to poorer survival, although the survival disparity between high and low SII was less strong compared to the non-NT cohort.

The combination of SII with LVI provided further insights into survival stratification. LVI has been well established as a marker of poor prognosis, and its combination with high SII increased survival disadvantage. In patients who did not receive NT, the combination of high SII and LVI+ resulted in the worst survival outcomes. On the other hand, patients with low SII and LVI- exhibited the best survival, confirming the dual prognostic role of both SII and LVI. The survival curve for patients with high SII and LVI+ was notably worse, emphasizing the negative impact of these factors on survival.

In the NT subgroup, the negative prognostic impact of high SII persisted, but the effect of LVI was reduced. While LVI+ combined with high SII still resulted in poorer survival, the survival differences between the subgroups with and without LVI were less pronounced compared to the non-NT cohort. Furthermore, when analyzing pre-treatment SII (SII_pre_NT) in patients who received NT, SII continued to show a clear association with survival. High pre-treatment SII correlated with worse survival regardless of LVI status. This highlights the potential role of SII as a biomarker for prognosis in patients undergoing NT, underscoring its importance even prior to treatment initiation.

In conclusion, SII was identified as a robust prognostic factor for survival, with high SII associated with poorer outcomes in both patients who received NT and those who did not. The combination of high SII with LVI+ further worsened survival, particularly in patients without NT. These findings suggest that both SII and LVI should be considered in clinical prognostication and treatment planning, particularly in the context of NT, where SII remains a significant independent factor influencing survival outcomes.

## 4. Discussion

Although LVI is a well-known adverse prognostic factor in gastrointestinal malignancies, including AEG [21,22], its prognostic relevance in combination with SII appears to diminish in patients undergoing NT. In this cohort, multivariable models excluding LVI consistently demonstrated comparable or better predictive performance, as assessed by AIC and IBS. One plausible explanation for the diminished role of LVI following NT is treatment-induced stromal fibrosis, which can obscure lymphovascular structures and harm histologic detection. NT regimens are known to provoke profound tissue remodelling, including fibrosis, inflammation, and vascular regression, potentially leading to underestimation of LVI. This phenomenon has been previously described in rectal [27], breast [28,29] and gastrointestinal cancer [30,31,32]. These results show the importance of standardized pathological assessment, including consideration of treatment-related artifacts that may compromise diagnostic accuracy. In contrast, among primarily resected patients, LVI retained its expected prognostic effect and showed additive value when combined with SII therefore being a potentially reliable biomarker in treatment context. Furthermore, LVI is known to suffer from substantial interobserver variability, which may have affected reproducibility. Future studies incorporating immunohistochemical validation and central pathology review may help mitigate this bias.

Although our findings are promising, several limitations must be considered. The retrospective, single-centre nature of the study limits generalizability and introduces potential selection bias. Additionally, SII may be influenced by non-malignant inflammatory conditions and LVI assessment is subject to interobserver variation. Sample size limitations, particularly in subgroup analyses, may have reduced the power to detect modest associations. In addition to that, recent multicentred studies and meta-analyses have increasingly highlighted the prognostic significance of both SII [33] and LVI [34] in esophageal cancer. However, many prior investigations suffer from institutional repetition and lack consensus regarding optimal cutoff values and standardized pathological evaluation.

From a clinical perspective, the combination of LVI and SII could potentially inform postoperative risk stratification. However, given the retrospective design and limited sample size, such implications must be interpreted with caution. This study is the first one to investigate the association of SII and LVI and therefore it underscores the necessity for prospective and multicentric validation studies. Such efforts are essential to overcome current limitations, enhance external validity, and potentially integrate these markers into personalized postoperative risk stratification and treatment decision algorithms. These findings contribute to this emerging literature by highlighting their interaction and context-dependent behaviour.

## 5. Limitations

This study has several limitations. Firstly, its retrospective design introduces inherent biases and restricts causal inference. Secondly, the single-center nature limits generalizability. Thirdly, histopathological assessments such as LVI are subject to inter-observer variability. Additionally, while SII is a reproducible and accessible biomarker, it may be influenced by non-cancer-related inflammatory states. Finally, the sample size, particularly in subgroup analyses, may limit the power to detect smaller effects.

## 6. Conclusions

These results highlight a significant association between SII and LVI in patients with resectable AEG. The combined presence of high SII and positive LVI correlated with notably worse survival outcomes, especially in patients who did not undergo NT. While SII alone remained a strong prognostic marker across all patient subgroups, LVI provided additional prognostic value primarily in those undergoing primary surgery. These results underscore the importance of considering both systemic inflammation and LVI in risk stratification and personalized treatment strategies for AEG.

## Figures and Tables

**Table 1:** Clinicopathological characteristics.

Characteristic		All Patients	Neoadjuvant Therapy	
		Overall (*n* = 211)	No (*n* = 137)	Yes (*n* = 74)
SII				
	Mean	783	784	781
	SD *	388	420	323
LVI, *n* (%)				
	-	116 (55%)	79 (58%)	37 (50%)
	+	95 (45%)	58 (42%)	37 (50%)
Age at surgery				
	Mean	65	66	62
	SD	11	11	10
Sex				
	Female	39 (18%)	26 (19%)	13 (18%)
	male	172 (82%)	111 (81%)	61 (82%)
(y)pT **, *n* (%)				
	0	1 (0.5%)	0 (0%)	1 (1.4%)
	1	38 (18%)	32 (23%)	6 (8.1%)
	2	69 (33%)	49 (36%)	20 (27%)
	3	93 (44%)	50 (36%)	43 (58%)
	4	10 (4.7%)	6 (4.4%)	4 (5.4%)
(y)pN, *n* (%)				
	0	82 (39%)	56 (41%)	26 (35%)
	1	90 (43%)	53 (39%)	37 (50%)
	2	25 (12%)	18 (13%)	7 (9.5%)
	3	14 (6.6%)	10 (7.3%)	4 (5.4%)
LK_ratio				
	Mean	0.21	0.21	0.20
	SD	0.26	0.26	0.25
	Unknown	3	3	0
G, *n* (%) ***				
	1	6 (2.8%)	5 (3.6%)	1 (1.4%)
	2	89 (42%)	59 (43%)	30 (41%)
	3	116 (55%)	73 (53%)	43 (58%)
Tumor type, *n* (%)				
	AEG I	115 (55%)	60 (44%)	55 (74%)
	AEG II	83 (39%)	71 (52%)	12 (16%)
	AEG III	13 (6.2%)	6 (4.4%)	7 (9.5%)
Surgery technique				
	Single-cavity	85 (40%)	70 (51%)	15 (20%)
	Two-cavity	126 (60%)	67 (49%)	59 (80%)
ASA preOP ****				
	1	45 (21%)	18 (13%)	27 (36%)
	2	139 (66%)	102 (74%)	37 (50%)
	3	24 (11%)	15 (11%)	9 (12%)
	4	3 (1.4%)	2 (1.5%)	1 (1.4%)
ECOG preOp *****				
	0	86 (43%)	55 (43%)	31 (43%)
	1	90 (45%)	57 (44%)	33 (46%)
	2	21 (10%)	15 (12%)	6 (8.3%)
	3	4 (2.0%)	2 (1.6%)	2 (2.8%)
	Unknown	10	8	2
UICC postOP				
	I	56 (27%)	43 (31%)	13 (18%)
	II	60 (28%)	37 (27%)	23 (31%)
	III	88 (42%)	50 (36%)	38 (51%)
	IV	7 (3.3%)	7 (5.1%)	0 (0%)
NT, *n* (%)		74 (35%)		
Mandard response				
	1	-	137 (100%)	0 (0%)
	2	-	0 (0%)	5 (6.8%)
	3	-	0 (0%)	16 (22%)
	4	-	0 (0%)	30 (41%)
	5	-	0 (0%)	23 (31%)
Chemotherapy regimen, *n* (%)				
	A	52 (25%)	0 (0%)	52 (70%)
	B	18 (8.5%)	0 (0%)	18 (24%)
	C	4 (1.9%)	0 (0%)	4 (5.4%)
	none	137 (65%)	137 (100%)	0 (0%)

Abbreviations: * SD standard deviation, ** p pathological staging, *** G tumor grading, **** ASA American society of anesthesiologists, ***** ECOG Eastern Cooperative Oncology Group Performance Status.

**Table 2:** The relationship between LVI and SII, stratified by treatment groups. LVI status is contrast-coded as − (no LVI) and + (presence of LVI).

		LVI			
Group	n	− ^1^	+ ^1^	*p*-Value ^2^	*p*-Superiority ^3^
SII (overall)	211	556 (367, 874)	924 (695, 1165)	<0.001	0.27 (0.21, 0.34)
SII (NT)	74	631 (399, 993)	816 (702, 1005)	0.021	0.34 (0.24, 0.47)
SII (no NT)	137	496 (339, 824)	1.013 (689, 1226)	<0.001	0.23 (0.17, 0.31)

^1^ Median (Q1, Q3), ^2^ Wilcoxon rank-sum test, ^3^ *p*-superiority (95% CI).

**Table 3:** Results of different Cox regression models for all patients. Regression coefficients (Estimate), hazard ratios (HR), standard errors (SE), z-values, and *p*-values from three separate Cox models: (1) model with LVI alone, (2) model with SII alone, and (3) combined model including both SII and LVI. The coefficient of determination (R^2^) is reported for each model.

Model Coefficients					
Covariate	Estimate	HR ^1^	SE ^2^	z-Value	*p*-Value
LVI (R^2^ = 0.128)					
LVI+	0.840	2.317	0.154	5.465	<0.001
SII (R^2^ = 0.441)					
SII	0.231	1.259	0.018	12.485	<0.001
SII + LVI (R^2^ = 0.485)					
SII	0.288	1.257	0.019	11.946	<0.001
LVI+	0.680	1.973	0.161	4.233	<0.001

^1^ Hazard ratio (HR), ^2^ Standard error (SE).

**Table 4:** Results of Cox regression models evaluating the prognostic impact of SII and LVI in patients undergoing primary resection. The table displays estimates, hazard ratios (HR), standard errors (SE), z-values, and *p*-values for three Cox models: (1) a model including LVI alone, (2) a model including SII alone, and (3) a combined model including both SII and LVI. The coefficient of determination (R^2^) indicates model fit.

Model Coefficients					
Covariate	Estimate	HR ^1^	SE ^2^	z-Value	*p*-Value
LVI (R^2^ = 0.192)					
LVI+	1.065	2.902	0.192	5.557	<0.001
SII (R^2^ = 0.451)					
SII	0.231	1.237	0.021	10.254	<0.001
SII + LVI (R^2^ = 0.524)					
SII	0.209	1.232	0.022	9.617	<0.001
LVI+	0.918	2.504	0.204	4.504	<0.001

^1^ Hazard ratio, ^2^ Standard error.

**Table 5:** Results of Cox regression models assessing the prognostic impact of SII and LVI in patients receiving neoadjuvant therapy (NT). The table presents regression coefficients (Estimate), hazard ratios (HR), standard errors (SE), z-values, and *p*-values for three Cox models: (1) LVI only, (2) SII only, and (3) both SII and LVI combined. Model performance is indicated by the coefficient of determination (R^2^).

Model Coefficients					
Covariate	Estimate	HR ^1^	SE ^2^	z-Value	*p*-Value
LVI (R^2^ = 0.030)					
LVI+	0.387	1.473	0.259	1.495	0.135
SII (R^2^ = 0.432)					
SII	0.335	1.398	0.052	6.421	<0.001
SII + LVI (R^2^ = 0.438)					
SII	0.337	1.401	0.053	6.330	<0.001
LVI+	0.233	1.262	0.2644	0.884	0.3771

^1^ Hazard ratio, ^2^ Standard error.

**Table 6:** AIC values of the best-performing Cox regression models from the selection for primarily resected patients. Shown are the AIC scores for models including different combinations of covariates. Lower AIC values indicate better model fit.

AIC	Used Parameters
LVI_SII	
785	SII, LVI, pT, pN, surgery_technique
786	SII, LVI, SEX, pT, pN, surgery_technique
786	SII, LVI, SEX, pT, pN, G, surgery_technique
SII	
785	SII, pT, pN, surgery_technique
785	SII, SEX, pT, pN, surgery_technique
785	SII, SEX, pT, pN, G, surgery_technique

**Table 7:** AIC values of top Cox regression models for patients receiving NT. Shown are the AIC scores for models including different combinations of covariates. Lower AIC values indicate better model fit.

AIC	Used Parameters
LVI_SII	
393	SII, LVI, age_at_OP, ECOG_pre_OP
394	SII, LVI, age_at_OP, pN, ECOG_pre_OP
394	SII, LVI, age_at_OP, pUICC, ECOG_pre_OP
SII	
391	SII, age_at_OP, ECOG_pre_OP
391	SII, age_at_OP, pN, ECOG_pre_OP
392	SII, age_at_OP, pUICC, ECOG_pre_OP

**Table 8:** Values of the parameters for the models from the selection for primarily resected patients.

		Model Coefficients			
Covariate	Estimate	HR	SE	z-Value	*p*-Value
LVI_SII					
SII	0.223	1.250	0.025	8.955	<0.001
LVI+	0.250	1.284	0.244	1.024	0.30
pT	0.402	1.494	0.141	2.849	0.004
pN	0.474	1.607	0.126	3.754	<0.001
Surgery_technique two-cavity	−0.404	0.668	0.201	−2.009	0.0456
SII					
SII	0.227	1.258	0.024	9.254	<0.001
pT	0.454	1.575	0.132	3.453	<0.001
pN	0.475	1.608	0.126	3.771	<0.001
Surgery_technique two-cavity	−0.440	0.644	0.198	−2.227	0.026

**Table 9:** Values of the parameters for the models from the selection for patients with NT.

		Model Coefficients			
Covariate	Estimate	HR	SE	z-Value	*p*-Value
LVI_SII					
SII	0.373	1.453	0.058	6.385	<0.001
LVI+	0.051	1.053	0.276	0.186	0.852
Age_at-OP	−0.026	0.974	0.014	−1.896	0.058
ECOG_pre_OP	0.497	1.644	0.202	2.465	0.014
SII					
SII	0.375	1.455	0.058	6.463	<0.001
Age_at-OP	−0.027	0.974	0.013	−1.980	0.048
ECOG_pre_OP	0.501	1.650	0.200	2.499	0.012

## Data Availability

Data are not available due to ethical restrictions.

## Abbreviations

The following abbreviations are used in this manuscript:

AEG	Adenocarcinoma of the Gastroesophageal Junction
AIC	Akaike Information Criterion
ASA	American Society of Anesthesiologists
CT	Computed Tomography
ECOG	Eastern Cooperative Oncology Group
GPS	Glasgow Prognostic Score
HR	Hazard Ratio
IBS	Integrated Brier Score
LVI	Lymphovascular Invasion
NLR	Neutrophil-to-Lymphocyte Ratio
NT	Neoadjuvant Therapy
PLR	Platelet-to-Lymphocyte Ratio
R^2^	Pseudo-R-squared (Cox & Snell)
SE	Standard error
SII	Systemic Immune-Inflammation Index
SII_pre_NT	Pre-treatment Systemic Immune-Inflammation Index
TRG	Tumor Regression Grade
UICC	Union for International Cancer Control

## Appendix B

**Table A1:** Patients characteristics according to LVI status and neoadjuvant therapy. Summary of clinical variables stratified by LVI presence-with (LVI+) and without (LVI-)-and NT status.

		Neoadjuvant Therapy			
		No		Yes	
Variables		LVI−	LVI+	LVI−	LVI+
SII	Mean	624	1001	712	851
	SD	343	420	368	256
mean Age * (SD)		65	67	64	60
Sex, *n* (%)					
	Male	65 (82%)	46 (79%)	31 (84%)	30 (81%)
	Female	14 (18%)	12 (21%)	6 (16%)	7 (19%)
(y)pT					
	0				1 (2.7%)
	1	29 (37%)	3 (5.2%)	5 (14%)	1 (2.7%)
	2	33 (42%)	16 (28%)	14 (38%)	6 (16%)
	3	15 (19%)	35 (60%)	16 (43%)	27 (73%)
	4	2 (2.5%)	4 (6.9%)	2 (5.4%)	2 (5.4%)
(y)pN					
	0	46 (58%)	10 (17%)	16 (43%)	10 (27%)
	1	27 (34%)	26 (45%)	17 (46%)	20 (54%)
	2	2 (2.5%)	16 (28%)	4 (11%)	3 (8.1%)
	3	4 (5.1%)	6 (10%)	0 (0%)	4 (11%)
	4	-	-	-	-
UICC postOP					
	I	41 (52%)	2 (3.4%)	10 (27%)	3 (8.1%)
	II	19 (24%)	18 (31%)	12 (32%)	11 (30%)
	III	16 (20%)	34 (59%)	15 (41%)	23 (62%)
	IV	3 (3.8%)	4 (6.9%)		
G, *n* (%)					
	1	4 (5.1%)	1 (1.7%)	1 (2.7%)	0 (0%)
	2	44 (56%)	15 (26%)	17 (46%)	13 (35%)
	3	31 (39%)	42 (72%)	19 (51%)	24 (65%)
Lymphnode ratio					
	Mean	0.11	0.35	0.13	0.28
	SD	0.17	0.29	0.22	0.26
	unknown	1	2		
Tumor type, *n* (%)					
	AEG I	40 (51%)	20 (34%)	27 (73%)	28 (76%)
	AEG II	34 (43%)	37 (64%)	6 (16%)	6 (16%)
	AEG III	5 (6.3%)	1 (1.7%)	4 (11%)	3 (8.1%)
Surgery technique					
	One-cavity	35 (44%)	35 (60%)	5 (14%)	10 (27%)
	Two-cavity	44 (56%)	23 (40%)	32 (86%)	27 (73%)
ASA					
	1	9 (11%)	9 (16%)	13 (35%)	14 (38%)
	2	63 (80%)	39 (67%)	16 (43%)	21 (57%)
	3	6 (7.6%)	9 (16%)	7 (19%)	2 (5.4%)
	4	1 (1.3%)	1 (1.7%)	1 (2.7%)	0 (0%)
ECOG					
	0	33 (43%)	22 (42%)	15 (41%)	16 (46%)
	1	37 (49%)	20 (38%)	16 (43%)	17 (49%)
	2	6 (7.9%)	9 (17%)	4 (11%)	2 (5.7%)
	3	0 (0%)	2 (3.8%)	2 (5.4%)	0 (0%)
Mandard response					
	2	-	-	3 (8.1%)	2 (5.4%)
	3	-	-	12 (32%)	4 (11%)
	4	-	-	15 (41%)	15 (41%)
	5	-	-	7 (19%)	16 (43%)
Chemotherapy regimen					
	A	-	-	27 (73%)	25 (68%)
	B	-	-	9 (24%)	9 (24%)
	C	-	-	1 (2.7%)	3 (8.1%)
	none	-	-		

Abbreviations: NT neoadjuvant therapy, SD standard deviation, c clinical staging, p pathological staging, ASA American society of anesthesiologists classification, ECOG Eastern Cooperative Oncology Group Performance Status, HR hazard ratio, CI confidential interval; * age at time of surgery

**Table A2:** Univariate Cox regression analyses estimating the influence of clinicopathologic parameters on OS in patients with primarily resected AEG and patients with neoadjuvant treatment (models for other variables of interest).

Variables		Primarily Resected			Neoadjuvant Therapy		
		HR	95% CI	*p*-Value	HR	95% CI	*p*-Value
Age (years)		1.01	0.99–1.03	0.302	1.00	0.98–1.02	0.998
SEX (ref. male)		0.93	0.59–1.47	0.756	1.49	0.73–3.043	0.271
G (ref. G3)		-	-	-	-	-	-
	2	1.17	0.42–3.24	0.769	0.20	0.03–1.56	0.125
	3	2.53	0.92–6.98	0.073	0.28	0.04–2.13	0.219
UICC_postOP							
	I	-	-		-	-	
	II	3.10	1.86–5.16	**<0.001**	1.19	0.56–2.56	0.649
	III	4.93	2.99–8.12	**<0.001**	1.23	0.60–2.51	0.568
	IV	3.27	1.41–7.60	**0.006**	-	-	-
(y)pT		1.90	1.53–2.37	**<0.001**	1.35	0.94–1.95	0.107
(y)pN		1.96	1.61–2.38	**<0.001**	1.23	0.87–1.76	0.243
Mandard response							
	2	-	-	-	-	-	-
	3		-	-	0.78	0.25–2.42	0.665
	4				0.99	0.34–2.87	0.985
	5				1.43	0.49–4.20	0.516
Lymph node ratio		22.4	10.2–48.9	**<0.001**	3.71	1.52–9.10	**0.004**
Mandard response		-	-	-	1.222	0.916–1.630	0.167
Tumor type							
	AEG I	-					
	AEG II	1.27	0.87–3.25	0.769	0.20	0.03–1.56	0.125
	AEG III	2.53	0.92–6.98	0.073	0.28	0.04–2.13	0.219
ASA_preOP		1.18	0.84–1.67	0.324	1.03	0.71–1.49	0.891
ECOG_preOP		1.23	092–1.64	0.170	1.27	0.93–1.74	0.132

Abbreviations: NI not included, c clinical staging, p pathological staging, OP operation, HR hazard ratio, CI confidence interval, ASA American society of anesthesiologists classification, ECOG Eastern Cooperative Oncology Group. Bold values indicate statistical significance.

## References

[1] Smyth E.C., Nilsson M., Grabsch H.I., van Grieken N.C., Lordick F. (2020). Gastric cancer. Lancet.

[2] Sung H., Ferlay J., Siegel R.L., Laversanne M., Soerjomataram I., Jemal A., Bray F. (2021). Global Cancer Statistics 2020: GLOBOCAN Estimates of Incidence and Mortality Worldwide for 36 Cancers in 185 Countries. CA A Cancer J. Clin..

[3] Arnold M., Soerjomataram I., Ferlay J., Forman D. (2015). Global incidence of oesophageal cancer by histological subtype in 2012. Gut.

[4] Morgan E., Arnold M., Rutherford M.J., Bardot A., Ferlay J., Moller H., Bray F., Soerjomataram I., Allemani C., Johnson C.J. (2021). International trends in oesophageal cancer survival by histological subtype between 1995 and 2014. Gut.

[5] Dhakras P., Uboha N., Horner V., Reinig E., Matkowskyj K.A. (2020). Gastrointestinal cancers: Current biomarkers in esophageal and gastric adenocarcinoma. Transl. Gastroenterol. Hepatol..

[6] Stotz M., Pichler M., Absenger G., Szkandera J., Arminger F., Schaberl-Moser R., Krenn-Pilko S., Gerger A., Kornprat P., Ressler S. (2013). Increased neutrophil-lymphocyte ratio is a poor prognostic factor in patients with primary operable and inoperable pancreatic cancer. Br. J. Cancer.

[7] Szkandera J., Pichler M., Stotz M., Absenger G., Arminger F., Weissmueller M., Alidzanovic L., Kornprat P., Stojakovic T., Gerger A. (2013). Elevated preoperative neutrophil/lymphocyte ratio is associated with poor prognosis in soft-tissue sarcoma patients. Br. J. Cancer.

[8] Jomrich G., Paireder M., Gleiss A., Kristo I., Harpain L., Schoppmann S.F. (2017). Comparison of Inflammation-Based Prognostic Scores in a Cohort of Patients with Resectable Esophageal Cancer. Gastroenterol. Res. Pract..

[9] Jomrich G., Paireder M., Gleiss A., Kristo I., Harpain L., Schoppmann S.F. (2018). The modified Glasgow prognostic score is an independent prognostic indicator in neoadjuvantly treated adenocarcinoma of the esophagogastric junction. Oncotarget.

[10] Jomrich G., Yan W., Kollmann D., Kristo I., Winkler D., Puhr H., Lhan-Mutlu A., Hollenstein M., Asari R., Schoppmann S.F. (2024). Elevated fibrinogen-albumin ratio is an adverse prognostic factor for patients with primarily resected gastroesophageal adenocarcinoma. J. Cancer Res. Clin. Oncol..

[11] Jomrich G., Hollenstein M., John A., Harpain L., Kristo I., Schoppmann S.F. (2021). High Systemic Immune-Inflammation Index is an Adverse Prognostic Factor for Patients With Gastroesophageal Adenocarcinoma. Ann. Surg..

[12] Salazar-Valdivia F.E., Ávila-Aguero M.L., Gil-Torres J., Santos A., Agüero D., Nitsch-Velásquez L. (2023). Systemic Immune-Inflammation Index and Mortality in Testicular Cancer: A Systematic Review and Meta-Analysis. Diagnostics.

[13] Islam M.M., Satici M.O., Eroglu S.E. (2024). Unraveling the clinical significance and prognostic value of the neutrophil-to-lymphocyte ratio, platelet-to-lymphocyte ratio, systemic immune-inflammation index, systemic inflammation response index, and delta neutrophil index: An extensive literature review. Turk. J. Emerg. Med..

[14] Hu B., Yang X.R., Xu Y., Sun Y.F., Sun C., Guo W., Zhang X., Wang Y., Qiu S.J., Shi J. (2014). Systemic Immune-Inflammation Index Predicts Prognosis of Patients after Curative Resection for Hepatocellular Carcinoma. Clin. Cancer Res..

[15] Jomrich G., Gruber E.S., Winkler D., Hollenstein M., Gasser R., Szkandera J., Kornprat P., Weissmueller M., Schoppmann S.F., Stotz M. (2020). Systemic Immune-Inflammation Index (SII) Predicts Poor Survival in Pancreatic Cancer Patients Undergoing Resection. J. Gastrointest. Surg..

[16] Ji Y., Wang H. (2020). Prognostic prediction of systemic immune-inflammation index for patients with gynecological and breast cancers: A meta-analysis. World J. Surg. Oncol..

[17] Alitalo K., Carmeliet P. (2002). Molecular mechanisms of lymphangiogenesis in health and disease. Cancer Cell.

[18] Sleeman J.P., Thiele W. (2009). Tumor metastasis and the lymphatic vasculature. Int. J. Cancer.

[19] Van Trappen P.O., Pepper M.S. (2002). Lymphatic dissemination of tumour cells and the formation of micrometastases. Lancet Oncol..

[20] Stacker S.A., Williams S.P., Karnezis T., Shayan R., Fox S.B., Achen M.G. (2014). Lymphangiogenesis and lymphatic vessel remodelling in cancer. Nat. Rev. Cancer.

[21] Schoppmann S.F., Jesch B., Zacherl J., Riegler M.F., Friedrich J., Birner P. (2013). Lymphangiogenesis and lymphovascular invasion diminishes prognosis in esophageal cancer. Surgery.

[22] Zheng C., Cui M., Liu J., Zhang Q., Zhao G., Li Y. (2020). Lymphovascular Invasion as a Prognostic Factor in Non-Metastatic Adenocarcinoma of Esophagogastric Junction After Radical Surgery. Cancer Manag. Res..

[23] Rice T.W., Patil D.T., Blackstone E.H. (2017). 8th edition AJCC/UICC staging of cancers of the esophagus and esophagogastric junction: Application to clinical practice. Ann. Cardiothorac. Surg..

[24] Mandard A.M., Dalibard F., Mandard J.C., Marnay J., Henry-Amar M., Petiot J.F., Roussel A., Jacob J.H., Segol P., Samama G. (1994). Pathologic assessment of tumor regression after preoperative chemoradiotherapy of esophageal carcinoma. Clinicopathologic correlations. Cancer.

[25] Siewert J.R., Stein H.J. (1998). Classification of adenocarcinoma of the oesophagogastric junction. Br. J. Surg..

[26] Ichhpuniani S., Jamal M., Deeb A.P., Khan T., Gahagan J., Kim D., Aragon-Ching J.B., Shah N., Catenacci D.V.T., Margolis C.A. (2024). Lymph Node Ratio as a Predictor of Survival for Colon Cancer: A Systematic Review and Meta-Analysis. Am. Surg..

[27] Hav M., Libbrecht L., Ferdinande L., Geboes K., Pattyn P., Cuvelier C.A. (2015). Pathologic Assessment of Rectal Carcinoma after Neoadjuvant Radio(chemo)therapy: Prognostic Implications. Biomed Res. Int..

[28] Thompson N., Storr S., Zhang S., Martin S. (2015). Lymphovascular invasion: Assessment and prognostic impact in melanoma and breast cancer. Histol. Histopathol..

[29] Tamura N., Hasebe T., Okada N., Houjoh T., Akashi-Tanaka S., Shimizu C., Shibata T., Sasajima Y., Iwasaki M., Kinoshita T. (2009). Tumor histology in lymph vessels and lymph nodes for the accurate prediction of outcome among breast cancer patients treated with neoadjuvant chemotherapy. Cancer Sci..

[30] Langer R., Becker K. (2018). Tumor regression grading of gastrointestinal cancers after neoadjuvant therapy. Virchows Arch..

[31] Zhou J., Yang Y., Zhang H., Luan S., Xiao X., Li X., Fang P., Gu Y., Chen L., Zeng X. (2023). Lymphovascular and Perineural Invasion After Neoadjuvant Therapy in Esophageal Squamous Carcinoma. Ann. Thorac. Surg..

[32] Thies S., Langer R. (2013). Tumor regression grading of gastrointestinal carcinomas after neoadjuvant treatment. Front Oncol..

[33] Zhang Y., Xiao G., Wang R. (2019). Clinical significance of systemic immune-inflammation index (SII) and C-reactive protein-to-albumin ratio (CAR) in patients with esophageal cancer: A meta-analysis. Cancer Manag. Res..

[34] Wang A., Tan Y., Geng X., Chen X., Wang S. (2019). Lymphovascular invasion as a poor prognostic indicator in thoracic esophageal carcinoma: A systematic review and meta-analysis. Dis. Esophagus.

